# Evaluation of Alterations in Nutrient Utilization and Intestinal Health in Response to Heat Stress in Pekin Ducks Based on a Pair-Feeding Experimental Design

**DOI:** 10.3390/ani15152213

**Published:** 2025-07-28

**Authors:** Xiangyi Zeng, Arshad Javid, Gregory S. Fraley, Gang Tian, Keying Zhang, Shiping Bai, Xuemei Ding, Jianping Wang, Yan Liu, Yue Xuan, Shanshan Li, Qiufeng Zeng

**Affiliations:** 1 Animal Nutrition Institute, Key Laboratory for Animal Disease-Resistance Nutrition of China Ministry of Education, Sichuan Agricultural University, Chengdu 611130, Chinatgang2008@126.com (G.T.);; 2 Department of Wildlife and Ecology, University of Veterinary & Animal Science, Lahore 54000, Pakistan; arshadjavid@uvas.edu.pk; 3 Animal Science Department, Purdue University, West Lafayette, IN 47907, USA

**Keywords:** duck, gut microbiota, heat stress, intestinal health, nutrient utilization, gene expression

## Abstract

The objective of this study was to investigate alterations in nutrient utilization, standardized ileal digestibility of amino acids (SIDAA), and intestinal health in response to heat stress (HS) in Pekin ducks. A total of 240 healthy 28-day-old male Pekin ducks were randomly allocated to three groups: a normal control (NC) group, an HS group, and a pair-fed (PF; provided an amount of feed equal to that consumed by the HS group to eliminate the effects of feed intake) group, each with eight replicate cages of ten birds. The results showed that HS significantly reduced the apparent utilization of dietary energy, ether extract, and crude protein compared to both the NC and PF groups (*p* < 0.05), but yielded comparable SIDAA to the PF group. The HS group exhibited reduced mRNA levels of *EAAT3* and *PepT1*, along with elevated mRNA levels of *CAT1*, *GLUT5*, and *FATP6* in the jejunum compared to the NC or PF groups, respectively (*p* < 0.05). Furthermore, HS resulted in a significant deterioration of jejunal morphology and goblet cell count compared to the NC and PF groups (*p* < 0.05). Serum fluorescein isothiocyanate-dextran levels were significantly higher in HS ducks than in NC ducks (*p* < 0.05), but did not differ from PF ducks. At order-level classification of ileal mucosal microbiota, HS markedly increased the relative abundance of *Bacillales*, *Deferribacterales*, and *Actinomycetales* versus NC (*p* < 0.05), while significantly decreasing *Bifidobacteriales* abundance relative to PF (*p* < 0.05). Collectively, HS induces a leaky gut and microbiota dysbiosis that compromises gut health, thereby reducing dietary nutrient utilization in Pekin ducks. The observed reduction in feed intake constitutes a primary driver of intestinal health deterioration in heat-stressed Pekin ducks.

## Simple Summary

As global temperatures rise, heat stress (HS) is becoming a prevalent stressor with deleterious consequences for duck growth and health. Under HS conditions, birds reduce feed intake (FI) and undergo physiological adaptations to mitigate heat production or enhance heat dissipation, yet HS effects on nutrient utilization and gut health in meat ducks—particularly under pair-fed experimental designs—remain poorly characterized. This study investigated alterations in nutrient utilization, standardized ileal amino acid digestibility, and intestinal health parameters in Pekin ducks exposed to HS. The results demonstrated that HS induces intestinal barrier dysfunction and microbiota dysbiosis, thereby impairing gut health and consequently reducing dietary nutrient utilization. Critically, although reduced FI constitutes an adaptive mechanism to limit metabolic heat load, data indicate this FI reduction represents a primary driver of intestinal health compromise in HS-exposed Pekin ducks.

## 1. Introduction

As global temperatures rise, heat stress (HS) has become a prevalent stressor with deleterious consequences for duck growth and health. Fast-growing ducks are particularly sensitive to HS due to their high stocking density, high metabolic rate, down feather coverage, and the absence of sweat glands [1]. Under HS conditions, birds typically decrease their feed intake (FI) and undergo a series of physiological alterations that reduce performance to decline heat production [2]. The use of pair-feeding techniques, where birds under thermoneutral conditions are pair-fed the same amount of feed as birds subjected to constant HS, has demonstrated that approximately 81% of the body weight gain (BWG) reduction was attributed to a reduction in FI, with the remaining 19% being the consequence of physiological changes induced by HS per se [3]. From a nutritional perspective, reduced nutrient digestibility and altered postabsorptive metabolism can impair the performance of heat-stressed birds. In our previous study, we reported the decrease in FI and BWG and the metabolic mechanisms underlying the suboptimal performance of Pekin ducks induced by HS [4]. Although FI influences nutrient digestion and absorption in animals [5,6], no studies have investigated the effects of HS on nutrient utilization in meat ducks using a pair-feeding experimental design.

As is well known, intestinal health is critically important for the digestion and absorption of nutrients in poultry [7]. Several studies have demonstrated that HS led to increased crypt depth and reduced villus height and villus height to crypt depth ratio in poultry [8,9,10], resulting in lower nutrient absorptive capacity [11]. The adverse effects of high ambient temperatures are also evident in gut leak, which allow pathogens and toxins present in the intestinal lumen to be translocated, ultimately leading to gut dysfunction [12]. Zhang et al. [13] confirmed that increased gut permeability in broilers under HS conditions was linked to reduced tight junction proteins and altered gut microbiota composition. In particular, gut microbiota could ferment indigestible fiber to produce a multitude of fermentation products, including short-chain fatty acids, that play a vital role in maintaining intestinal barrier function [14]. Nevertheless, the effects of HS on gut microbiota in meat ducks remain largely unexplored. Therefore, in our current study, changes in nutrient utilization and intestinal health in response to HS in Pekin ducks were further interpreted using a metabolic trial, morphological analyses, qRT-PCR and 16S rRNA sequencing. We postulated that HS would have adverse effects on intestinal health, as evidenced by the impairment of jejunal morphology and the alteration of ileal microbiota composition, which reduced intestinal nutrient digestible and absorptive capacity in Pekin ducks. The research results can provide a theoretical basis for mitigating HS-induced intestinal damage in meat ducks through nutritional regulation.

## 2. Materials and Methods

### 2.1. Birds, Diets, and Management

This experiment was conducted in the Academic and Research Zone of Ya’an Campus, Sichuan Agricultural University. A total of 240 healthy 28-day-old male Pekin ducks (2054 ± 28 g) were allotted randomly to the following three groups with 8 replicate cages of 10 birds each: the normal control (NC) group at constant 21 ± 1 °C, the HS group at 34 ± 1 °C for 7 h daily with relative humidity (RH) between 50% and 60% and the rest of the day at 25 ± 1 °C, and the pair-fed (PF) group at constant 21 ± 1 °C (in the same house as the ducks in the NC group). The ducks were provided with the same amount of feed as the HS group on the previous day. Temperature and humidity were monitored using hygrothermographs (Tianjin Kehui Instrument Factory, Tianjin, China) mounted above each cage. Each replicate group was housed in a single cage (1.0 × 0.8 × 0.6 m) under a 16 h light/8 h dark cycle. All birds received a starter and a finisher diet, the composition and nutrient density of which are listed in Table 1 and Table 2, respectively, in accordance with our previous research [4]. In our previous study, Pekin ducks subjected to HS conditions (34 ± 1 °C, 7 h/d, 14 d) increased their panting frequency and rectal temperature, as well as the serum concentration of heat shock protein 70 and corticosterone, suggesting that the chronic HS model was successfully established [4]. Therefore, we further evaluated the effect of HS on intestinal health and dietary nutrient utilization and standardized ileal digestibility of amino acids (SIDAA) in this study.

### 2.2. Intestinal Permeability Determination

On day 42, one duckling per replicate was selected to determine intestinal permeability using fluorescein isothiocyanate dextran (FITC-d, 4kDa, Sigma, Ronkonkoma, NY, USA), an indicator to examine barrier function. All birds were administered FITC-d orally (2.2 mg per duck), and then blood was collected from the jugular vein at two hours post-FITC-d administration. Serum FITC-d levels were quantified at excitation and emission wavelengths of 485 and 528 nm, respectively (BioTek Instruments, Winooski, VT, USA). Subsequently, serum FITC-d concentration was calculated based on a standard curve with known FITC-d levels.

### 2.3. Jejunal Morphology Examination

On day 42, one duckling per replicate was humanely euthanized through exsanguination for jejunal morphology analysis, gene expression assays, and 16S rRNA sequencing. A fixed jejunal segment was embedded in 10% paraffin, sectioned into 5 μm slices, and stained with either hematoxylin and eosin (H&E) to evaluate intestinal architecture or Alcian blue (AB) to quantify the number of goblet cells. Images were captured using a microscope (BA400 Digital, Xiamen, China) and analyzed using Image-Pro Plus 6.0 (Media Cybernetics, Rockville, MD, USA) to determine villus height (VH), crypt depth (CD), and their ratio (VH/CD). Goblet cells along the villus were counted by light microscopy.

### 2.4. Gene Expression Assays

Total RNA was extracted from frozen jejunal mucosa samples using a Trizol reagent (TaKaRa, Dalian, China), and first-strand cDNA synthesis was performed with the PrimeScript™ RT Reagent Kit (Takara, China) in accordance with the manufacturer’s instructions. The quantitative real-time PCR (qRT-PCR) was performed on the ABI QuantStudio™ 6 Flex system (Applied Biosystems, Waltham, MA, USA). The primer sequences for the target genes were designed using the National Centre for Biotechnology Information (NCBI) Blast tool. All primers set in the qRT-PCR reaction were run for melting curve analyses to generate a standard curve to assess PCR efficiency. A comprehensive list of all primer sequences used in this study is presented in Table 3. Relative gene expression was quantified by normalizing to the expression of β-actin according to the 2^−ΔΔCt^ method, with the quantity of the NC group scaled to approximately 1.

### 2.5. 16S rRNA Gene Sequencing

Total genome DNA from the samples was extracted using the cetyltrimethylammonium bromide method, and the concentration and purity of DNA were detected using a 1% agarose gel. Subsequently, PCR amplification and product purification were performed sequentially. Sequencing libraries were generated using the TruSeq^®^ DNA PCR-Free Sample Preparation Kit (Illumina, San Diego, CA, USA) in accordance with the manufacturer’s instructions, and library quality was assessed on the Qubit@ 2.0 Fluorometer (Thermo Scientific, Waltham, MA, USA) and Agilent Bioanalyzer 2100 systems. The samples were then sequenced using the Illumina NovaSeq 6000 platform, and high-quality clean tags were obtained according to the QIIME 2 quality-controlled process. The representative sequences of Operational Taxonomic Units (OTUs) were selected, and OTU clustering was performed based on the principle of 97% sequence similarity.

### 2.6. Assay of Nutrient Utilization and Standard Ileal Digestibility of Amino Acids

On day 43, two ducks randomly selected from each replicate were housed in metabolic cages (two ducks per cage) and assigned to the original groups. In addition, this experiment included two nitrogen-free diet groups under thermoneutral and HS conditions, with eight replicates per group. All ducks were fed the original diets or the nitrogen-free diet supplemented with 0.5% titanium dioxide (TiO_2_) as an indigestible marker. Excreta samples were collected from each cage over a period of 72 h. Following the removal of any debris, the samples were gathered in each cage and dried in an oven at 65 °C for 3 days. All samples were ground to pass through a 0.5 mm screen and then analyzed for dry matter (DM), gross energy (GE), ether extract (EE), nitrogen (N), calcium (Ca), and total phosphorus (TP) in accordance with the method (AOAC, 2005) [15]. Crude protein (CP) was calculated as N × 6.25. EE in the diets and excreta was measured with a Soxhlet apparatus for approximately 8 h. GE was analyzed by using Parr 6400 oxygen bomb calorimeter (Parr Instrument Co., Moline, IL, USA). The TiO_2_ content in feed and excreta samples was measured according to the method proposed by Short et al. [16]. Nutrient utilization for the experimental diets was calculated with the following formula: Nutrient utilization (%) = {1 − [(N_e_ × T_d_)/(N_d_ × T_e_)]} × 100, where T_e_ = TiO_2_ concentration in excreta (% dry matter, DM), T_d_ = TiO_2_ concentration in the diet (% DM), N_e_ = nutrient concentration in excreta (% DM), and N_d_ = nutrient concentration in the diet (% DM).

Following the 72 h period of excreta collection on day 46, ducks were fed for 4 h, and then euthanized with carbon dioxide (CO_2_). Ileal digesta was collected from two birds per cage, pooled, and freeze-dried at −50 °C for three days to allow for subsequent analyses of TiO_2_ and amino acids (AAs). The AA contents were analyzed with an automatic amino acid analyzer (L-8900, HITACHI, Tokyo, Japan), in accordance with the method as described by Zhang et al. [17]. These data were used to calculate SIDAA based on our previous study [18].

### 2.7. Statistical Analyses

Data were subjected to Shapiro–Wilk and Levene’s tests to assess for normal distribution and variance homogeneity, respectively, with the SAS 9.4 software [19]. Two-tailed unpaired *t*-test or Mann–Whitney U test for normally or non-normally distributed datasets, respectively, was employed to ascertain statistical differences in parameters between the HS and control groups (NC and PF). The probability of *p* < 0.05 was considered to be statistically significant, and 0.05 < *p* < 0.1 was considered a trend. Data are shown as the mean ± standard error.

## 3. Results

### 3.1. Nutrient Utilization

As shown in Table 4, HS notably reduced the apparent utilization of dietary energy, EE, and CP, as well as apparent metabolizable energy (AME) when compared to the NC and PF groups (*p* < 0.05). Furthermore, the apparent utilization of dietary DM, TP, and Ca in the HS group was significantly lower than that in the PF group (*p* < 0.05).

### 3.2. Standardized Ileal Digestibility of Amino Acids

The Standardized Ileal Digestibility values of eight non-essential amino acids (Asp, Ser, Glu, Gly, Ala, Cys, Tyr, and Pro), seven essential amino acids (Thr, Val, Ile, Leu, Phe, His, and Arg), total non-essential amino acids (Total NEAAs), total essential amino acids (Total EAAs), and total amino acids were significantly higher in the HS group compared with those in the NC group (*p* < 0.05, Table 5). It was observed that ducks in the HS group had a tendency to increase the SID of Tyr in comparison to the PF group (*p* = 0.053).

### 3.3. Nutrient Transporter Gene Expression

**Figure 1:**
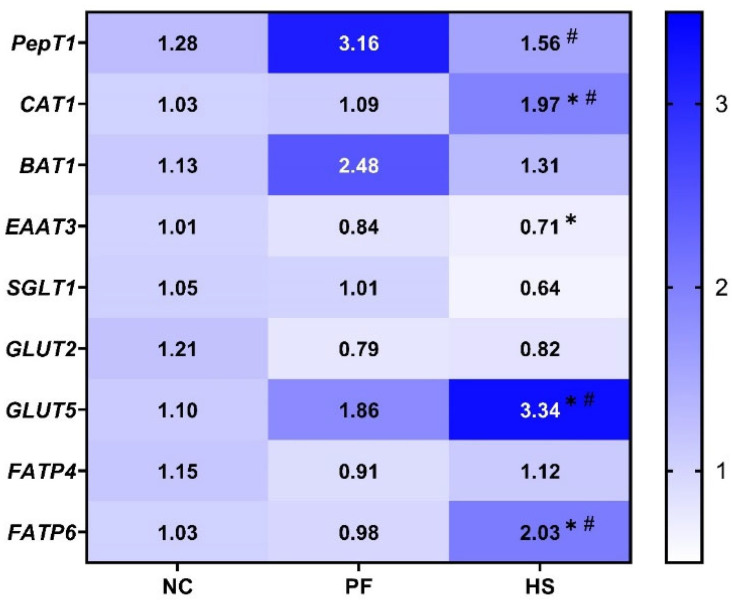
The effects of heat stress on mRNA expression of nutrient transporter genes in the jejunal mucosa of meat ducks; HS: heat stress group; PF: pair-fed group; NC: normal control group; * and ^#^ denote significant differences between HS and NC or PF at *p* < 0.05, respectively.

A reduction in the mRNA levels of excitatory amino acid transporters 3 (*EAAT3*) and oligopeptide transporter 1 (*PepT1*) was observed in the HS group in comparison to the NC and PF groups, respectively (*p* < 0.05, Figure 1). Nevertheless, HS notably up-regulated the mRNA expression of cationic amino acid transporter 1 (*CAT1*), glucose transporter 5 (*GLUT5*), and fatty acid transporter protein 6 (*FATP6*) compared to the NC and PF groups (*p* < 0.05).

### 3.4. Intestinal Permeability and Morphology

**Figure 2:**
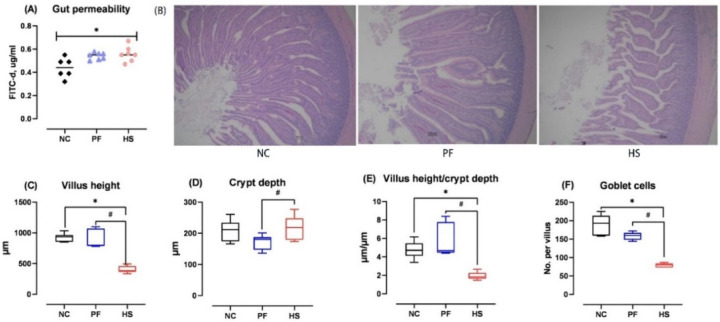
The effects of heat stress on gut permeability (**A**), jejunal morphology (**B**–**E**), and goblet cell number (**F**) of meat ducks; HS: heat stress group; PF: pair-fed group; NC: normal control group; * and ^#^ denote significant differences between HS and NC or PF at *p* < 0.05, respectively.

The serum FITC-d concentration of ducks in the HS group was significantly higher than that in the NC group (*p* < 0.05, Figure 2A), but was similar to that in the PF group. The jejunal morphology of ducks under HS conditions was impaired, as evidenced by lower VH, VH/CD, and goblet cell count, in addition to higher CD when compared to the NC and PF groups (*p* < 0.05, Figure 2B–F).

### 3.5. Changes in Ileal Mucosal Microbiota

**Figure 3:**
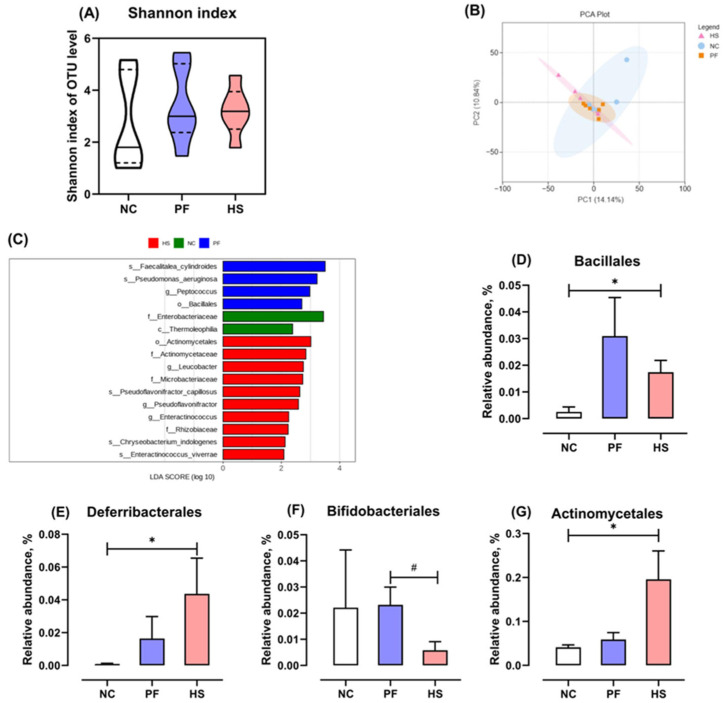
The effects of heat stress on the ileal mucosal microbiota in meat ducks: Shannon index (**A**), principal component analysis (**B**), linear discriminant analysis effect size (**C**), and the relative abundance of *Bacillales*, *Deferribacterales*, *Bifidobacteriales*, and *Actinomycetales* (**D**–**G**); HS: heat stress group; PF: pair-fed group; NC: normal control group; * and ^#^ denote significant differences between HS and NC or PF at *p* < 0.05, respectively.

The effects of HS on the microbiota in the ileal mucosa are illustrated in Figure 3. The Shannon index in the HS group was not significantly different compared to that in the NC and PF groups (*p* > 0.05, Figure 3A). Principal component analysis (PCA) demonstrated that the samples in the HS group did not form a distinct cluster that was clearly separated from those in the NC and PF groups (Figure 3B). The specific taxa in the microbiota of ileal mucosa that were significantly associated with HS were identified by Linear discriminant analysis Effect Size (LEfSe ≥ 2, family level). *Actinomycetaceae*, *Microbacteriaceae*, and *Rhizobiaceae* were notably enriched in the HS group, while *Enterobacteriaceae* was significantly enriched in the NC group (Figure 3C). More specifically, HS increased the relative abundance of *Bacillales*, *Deferribacterales*, and *Actinomycetales* at the order level compared to the NC group, whereas it notably reduced the relative abundance of *Bifidobacteriales* in comparison to the PF group (*p* < 0.05, Figure 3D–G).

## 4. Discussion

Several studies have reported that the apparent utilization of DM, CP, and energy in the diet were significantly reduced in heat-stressed broilers compared to those under thermoneutral conditions [20,21]. In a study by Bonnet et al. [22], which excluded the impact of FI by using pair-feeding techniques, it was observed that HS also decreased the apparent utilization of dietary DM, CP, and EE in broilers. In accordance with these previous studies, our findings suggest that HS led to a reduction in the apparent utilization of dietary DM, energy, EE, CP, Ca, and TP. Wallis and Balnave [23] observed a slight decrease in the apparent ileal digestibility (AID) for Thr, Ala, Met, Ile, and Leu under HS conditions (31 °C, 24 h/d, 29 d). Teyssier et al. [2] revealed the detrimental effect of HS (35 °C, 24 h/d, 21 d) on AA digestibility, with an average reduction of 5% in AID of total AAs. However, there was a tendency for the SID of Tyr to increase in the HS group compared to the PF group in this study. This difference may be due to the fact that ducks have a more developed digestive tract compared to chickens [24,25], and thus HS exerts less impact on the SIDAA of meat ducks. In our experimental setting (34 ± 1 °C, 7 h/d, 14 d), heat-stressed ducks exhibited slightly higher SIDAA in accordance with an elevated *CAT1* mRNA level in the jejunal mucosa, potentially compensating for enhanced AA requirements. Numerous studies have demonstrated significant upregulation of amino acid and small peptide transporters in the small intestine of heat-stressed broilers and swine [11,26]. In the present study, we observed marked upregulation of the CAT1 compared to both the PF and NC groups. This phenomenon exhibited a consistent trend with GLUT5 upregulation, potentially serving as a compensatory mechanism to address glucose supply deficits in heat-stressed meat ducks [4]. The maintenance of SIDAA under HS is attributed to the combined effects of reduced FI and intestinal compensatory mechanisms, with adaptive changes in the gut playing the predominant role. However, we found that HS resulted in reduced CP utilization in the diet. This paradoxical phenomenon may be partially attributed to hindgut damage causing increased endogenous nitrogen loss or microbiota dysbiosis reducing protein fermentation during the HS condition, thereby reducing the apparent utilization of CP.

In a study by He et al. [27], it was demonstrated that HS negatively affected intestinal morphology, resulting in a reduction in VH and VH/CD in the jejunum and ileum of ducks. Similarly, the results of our study showed that HS impaired jejunal morphology of ducks, as illustrated by reduced VH and VH/CD, as well as increased CD. The intestinal epithelia are covered with mucus secreted by goblet cells that can prevent intestinal mucosa from pathogen attacks and environmental toxins [8]. In the present study, HS significantly reduced jejunal goblet cell count, in agreement with the findings of Liu et al. [8] and Zhang et al. [28]. Further, FITC-d has been used as an indicator for the assessment of intestinal paracellular permeability, and the elevated concentration of FITC-d in serum implies impaired intestinal barrier function [29]. In the present study, the serum FITC-d levels of the HS group were found to be notably higher than those of the NC group, but similar to those of the PF group. Gilani et al. [30] showed that both a 4.5 h and a 9 h feed restriction period led to increased FITC-d concentrations in the blood of broilers. These findings indicate that HS may disrupt the intestinal barrier function in ducks by reducing FI. As demonstrated by Koch’s research, HS (Temperature–Humidity Index = 76) directly induced jejunal barrier damage in bovines compared to the PF control [31]. However, this effect was not observed in the current experiment, suggesting that triggering such responses may require either prolonged exposure duration or elevated temperature conditions.

Gut microbiota plays an indispensable role in intestinal mucosal homeostasis and gut health [32]. Several studies observed higher alpha diversity indices of microbiota in ileal contents and mucosal scrapings in chickens raised under high ambient temperature (HT) compared to thermoneutral conditions. However, our results showed that HS did not influence the alpha diversity of the microbiota in the ileal mucosa of ducks. Patra and Kar [33] highlighted that the effect of HT on the alpha diversity of gastrointestinal tract microbiota is contingent upon the duration and intensity of heat exposure. Previously, it was observed that the microflora structure of ileal content in chickens was altered by HT, based on unweighted UniFrac distance metric matrices [34]. Nevertheless, the results of our experiment in ducks demonstrated that HS had no effect on the microbiota structure in the ileal mucosa, in accordance with the findings of [35]. Xing et al. [36] posited that HT had a significant impact on the microbial structure of laying hens, which was primarily associated with a reduction in FI. The discrepancies observed in the aforementioned studies may be partly explained by the fact that intestinal mucosal flora is not easily affected by FI. The relative abundance of *Deferribacterales* was found to be significantly elevated in both mouse [37] and porcine [38] models suffering from inflammatory bowel disease and may release lipopolysaccharides to trigger inflammatory injuries and aggravate energy metabolism abnormalities [39]. In this study, we observed that HS markedly increased the relative abundance of *Deferribacterales*, whereas it notably reduced the relative abundance of *Bifidobacteriales*. Similarly, previous studies reported that HS decreased the populations of *Bifidobacterium* in the jejunal digesta of broilers [28,40]. These results suggest that HS increased harmful bacteria but decreased beneficial bacteria in the ileal mucosa at the order level.

## 5. Conclusions

Collectively, chronic HS-induced intestinal morphological injury, heightened intestinal permeability, and ileal mucosal microbiota dysbiosis contributed to reduced dietary nutrient utilization in Pekin ducks. The detrimental impact of FI reduction on intestinal health underscores FI regulation as a promising nutritional strategy to alleviate HS challenges in meat duck production. These insights into HS-driven interplay between gut health and nutrient utilization hold relevance for broader poultry species amid climate change, though strategy adaptation will be necessary to account for species-specific thermotolerance and intestinal physiology.

## Figures and Tables

**Table 1:** The composition and nutrition levels of the experimental diet in the starter phage (air dry basis).

Ingredients	Composition, %
Corn	57.80
Soybean meal	32.90
Wheat bran	2.88
Soybean oil	1.00
Dicalcium phosphate	1.70
Calcium carbonate	1.15
Sodium chloride	0.30
Choline chloride (50%)	0.15
L-Lysine HCL	0.21
L-Threonine	0.11
DL-Methionine	0.27
Vitamin premix ^1^	0.03
Mineral premix ^2^	0.50
Rice bran	1.00
Total	100.00
Calculated nutrients	
ME, kcal/kg	2900
Crude protein, %	19.50
Calcium, %	0.90
Non-phytate phosphorus, %	0.42
Digestible lysine, %	1.10
Digestible methionine, %	0.53
Digestible threonine, %	0.75

^1^ Vitamin premix provides the following per kg of final diet: vitamin A 8000 IU; vitamin D_3_ 2000 IU; vitamin E 5 mg; vitamin K_3_ 1.5 mg; vitamin B_1_ 0.6 mg; vitamin B_2_ 4.8 mg; vitamin B_6_ 1.8 mg; vitamin B_12_ 0.009 mg; niacin 10.5 mg; DL-calcium pantothenate 7.5 mg; folic acid 0.15 mg. ^2^ Mineral premix provides the following per kg of final diet: Fe (FeSO_4_·H_2_O) 80 mg; Cu (CuSO_4_·5H_2_O) 10 mg; Mn (MnSO_4_·H_2_O) 100 mg; Zn (ZnSO_4_·H_2_O) 60 mg; I (KI) 0.45 mg; Se (Na_2_SeO_3_) 0.3 mg.

**Table 2:** The composition and nutrition levels of the experimental diet in the finisher phage (air dry basis).

Ingredients	Composition, %
Corn	63.74
Soybean meal	26.00
Wheat bran	3.70
Soybean oil	1.20
Dicalcium phosphate	1.60
Calcium carbonate	1.13
Sodium chloride	0.30
Choline chloride (50%)	0.15
L-Lysine HCL	0.09
L-Threonine	0.04
DL-Methionine	0.23
Vitamin premix ^1^	0.03
Mineral premix ^2^	0.50
Rice bran	1.30
Total	100.00
Calculated nutrients	
ME, kcal/kg	2910
Crude protein, %	17.50
Calcium, %	0.85
Non-phytate phosphorus, %	0.40
Digestible lysine, %	0.85
Digestible methionine, %	0.46
Digestible threonine, %	0.60

^1^ Vitamin premix provides the following per kg of final diet: vitamin A 8000 IU; vitamin D3 2000 IU; vitamin E 5 mg; vitamin K3 1.5 mg; vitamin B1 0.6 mg; vitamin B2 4.8 mg; vitamin B6 1.8 mg; vitamin B12 0.009 mg; niacin 10.5 mg; DL-calcium pantothenate 7.5 mg; folic acid 0.15 mg; ^2^ Mineral premix provides the following per kg of final diet: Fe (FeSO_4_·H_2_O) 80 mg; Cu (CuSO_4_·5H_2_O) 10 mg; Mn (MnSO_4_·H_2_O) 100 mg; Zn (ZnSO_4_·H_2_O) 60 mg; I (KI) 0.45 mg; Se (Na_2_SeO_3_) 0.3 mg.

**Table 3:** Primers used for quantitative real-time PCR.

Target Genes ^1^	Primers	Sequences (5′–3′)	Fragment (bp)	Accession Number
	Reverse	AACTCCCACTGGCAGGAATG		
*PepT1*	Forward	GACGTTTGAGACTTCCCGGT	138	NM_001310803.1
	Reverse	AACTGAGCGTCAATGCCTGA		
*CAT1*	Forward	CATTTCCCTCTAGGGCCAGC	84	NM_001310833.1
	Reverse	CGGGGGATGAACTAAGAGCA		
*BAT1*	Forward	CTGAAAGGCGTTGGCATAGC	145	NM_001310802.1
	Reverse	TGTTGGCAGTGACAGGACAG		
*EAAT3*	Forward	GGGCTCGTATTCTGATGGCA	104	NM_001310783.1
	Reverse	CCACAAGCACTTGGCCTTTC		
*SGLT1*	Forward	TCTTCTGCAAGCGGGTCAAT	72	XM_005026696.5
	Reverse	TGCTGAGTCCAACTAGCAGC		
*GLUT2*	Forward	AAGTCTCCTTTCGCACCACC	139	XM_013099577.3
	Reverse	CCATTTCTTCCTGAGCCCGT		
*GLUT5*	Forward	CACAGGAACACCTGAGAGCG	71	XM_005015431.5
	Reverse	GTCCTGCGCGTCTTTTTGTC		
*FATP4*	Forward	AATCAGTACGTGGGGCTGTG	132	XM_027470622.2
	Reverse	CCCAAAAATGACCGCCTTGG		
*FATP6*	Forward	CATGTGTGGTTTGGACTCGC	94	XM_021272371.3
	Reverse	AGCTACTGAGACAGTGCAGG		
*β-actin*	Forward	ATGTCGCCCTGGATTTCG	165	GI151176138
	Reverse	CACAGGACTCCATACCCAAGAA		

^1^ PepT1: oligopeptide transporter 1; CAT1: cationic amino acid transporter 1; BAT1: bidirectional amino acid transporter 1; EAAT3: excitatory amino acid transporters 3; SGLT1: sodium-dependent glucose transporter 1; GLUT2: glucose transporter 2; GLUT5: glucose transporter 5; FATP4: fatty acid transporter protein 4; FATP6: fatty acid transporter protein 6; β-actin: Actin Beta.

**Table 4:** The effects of heat stress on nutrient utilization of meat ducks ^1^.

Items ^2^	NC ^3^	PF	HS	*p*-Value	
				HS vs. NC	HS vs. PF
DM, %	74.53 ± 0.76	74.75 ± 0.64	73.19 ± 1.64	0.071	0.047
Energy, %	78.79 ± 1.13	78.44 ± 0.63	76.42 ± 1.34	0.003	0.002
AME, kcal/kg	3054 ± 43.73	3041 ± 24.38	2962 ± 51.86	0.003	0.002
EE, %	88.59 ± 1.24	89.36 ± 3.00	84.55 ± 2.33	0.002	0.005
CP, %	60.21 ± 3.83	61.76 ± 1.10	48.51 ± 4.11	0.000	0.000
TP, %	57.40 ± 1.21	60.07 ± 2.36	54.51 ± 4.26	0.129	0.007
Ca, %	50.43 ± 4.03	60.95 ± 5.28	51.18 ± 7.63	0.824	0.012

^1^ Each value represents the mean value of 8 replicates/treatment (*n* = 8); ^2^ DM: dry matter; AME: apparent metabolizable energy; EE: ether extract; CP: crude protein; TP: total phosphorus; Ca: calcium; ^3^ NC: normal control group; PF: pair-fed group; HS: heat stress group.

**Table 5:** The effects of heat stress on standard ileal digestibility of amino acids of meat ducks ^1^.

Items ^2^, %	NC ^3^	PF	HS	*p*-Value	
				HS vs. NC	HS vs. PF
Asp	71.98 ± 3.60	76.76 ± 6.82	78.76 ± 1.74	0.001	0.514
Ser	78.28 ± 2.31	81.44 ± 6.73	85.04 ± 2.08	0.000	0.259
Glu	77.85 ± 3.12	82.58 ± 5.29	83.46 ± 1.38	0.002	0.708
Gly	64.70 ± 4.08	70.53 ± 8.88	73.32 ± 2.39	0.000	0.486
Ala	73.75 ± 2.88	77.79 ± 7.42	78.97 ± 3.84	0.013	0.737
Cys	78.08 ± 4.16	84.81 ± 5.19	87.73 ± 7.59	0.010	0.455
Tyr	78.43 ± 3.43	83.42 ± 4.90	88.18 ± 2.00	0.000	0.053
Pro	81.37 ± 2.93	83.89 ± 4.84	85.66 ± 2.47	0.014	0.444
Total NEAAs	75.74 ± 3.07	80.25 ± 6.07	82.15 ± 1.78	0.001	0.490
Thr	69.58 ± 4.24	74.97 ± 7.82	77.61 ± 3.43	0.003	0.467
Val	72.21 ± 3.19	76.87 ± 7.09	78.83 ± 3.44	0.003	0.556
Met	94.43 ± 3.35	96.78 ± 3.08	97.67 ± 2.00	0.058	0.565
Ile	75.41 ± 3.44	80.84 ± 6.32	83.43 ± 2.25	0.000	0.379
Leu	78.51 ± 3.07	82.60 ± 5.58	83.73 ± 2.02	0.004	0.659
Phe	79.10 ± 3.35	82.07 ± 5.67	82.58 ± 2.09	0.014	0.843
Lys	72.51 ± 5.08	76.96 ± 8.96	77.92 ± 5.09	0.072	0.824
His	77.95 ± 3.47	81.98 ± 5.83	83.74 ± 2.42	0.005	0.509
Arg	78.03 ± 2.41	82.69 ± 5.78	83.85 ± 3.18	0.003	0.675
Total EAAs	76.85 ± 3.35	81.12 ± 6.24	82.55 ± 2.76	0.005	0.619
Total AAs	76.11 ± 3.06	80.53 ± 6.17	82.22 ± 2.21	0.001	0.549

^1^ Each value represents the mean value of 8 replicates/treatment (*n* = 8); ^2^ Asp: aspartic acid; Ser: serine; Glu: glutamic acid; Gly: glycine; Ala: alanine; Cys: cysteine; Tyr: tyrosine; Pro: proline; Thr: threonine; Val: valine; Met: methionine; Ile: isoleucine; Leu: leucine; Phe: phenylalanine; Lys: lysine; His: histidine; Arg: arginine; NEAAs: non-essential amino acids; EAAs: essential amino acids; AAs: amino acids; ^3^ NC: normal control group; PF: pair-fed group; HS: heat stress group.

## Data Availability

Data are available upon request from the corresponding author.

## References

[1] Deeb N., Shlosberg A., Cahaner A. (2002). Genotype-by-environment interaction with broiler genotypes differing in growth rate. 4. Association between responses to heat stress and to cold-induced ascites. Poult. Sci..

[2] Teyssier J.R., Cozannet P., Greene E., Dridi S., Rochell S.J. (2023). Influence of different heat stress models on nutrient digestibility and markers of stress, inflammation, lipid, and protein metabolism in broilers. Poult. Sci..

[3] Teyssier J.R., Preynat A., Cozannet P., Briens M., Mauromoustakos A., Greene E.S., Owens C.M., Dridi S., Rochell S.J. (2022). Constant and cyclic chronic heat stress models differentially influence growth performance, carcass traits and meat quality of broilers. Poult. Sci..

[4] Zeng X., Javid A., Tian G., Zhang K., Bai S., Ding X., Wang J., Lv L., Xuan Y., Li S. (2024). Metabolomics analysis to interpret changes in physiological and metabolic responses to chronic heat stress in Pekin ducks. Sci. Total Environ..

[5] Shi F.Y., Guo N., Degen A.A., Niu J.H., Wei H.Y., Jing X.P., Ding L.M., Shang Z.H., Long R.J. (2020). Effects of level of feed intake and season on digestibility of dietary components, efficiency of microbial protein synthesis, rumen fermentation and ruminal microbiota in yaks. Anim. Feed Sci. Technol..

[6] Findeisen E., Südekum K.H., Fritz J., Hummel J., Clauss M. (2021). Increasing food intake affects digesta retention, digestibility and gut fill but not chewing efficiency in domestic rabbits (*Oryctolagus cuniculus*). J. Exp. Zool. Part A.

[7] Ducatelle R., Goossens E., Eeckhaut V., Van Immerseel F. (2023). Poultry gut health and beyond. Anim. Nutr..

[8] Liu L., Fu C., Yan M., Xie H., Li S., Yu Q., He S., He J. (2016). Resveratrol modulates intestinal morphology and HSP70/90, NF-κB and EGF expression in the jejunal mucosa of black-boned chickens on exposure to circular heat stress. Food Funct..

[9] Yi D., Hou Y., Tan L., Liao M., Xie J., Wang L., Ding B., Yang Y., Gong J. (2016). N-acetylcysteine improves the growth performance and intestinal function in the heat-stressed broilers. Anim. Feed Sci. Technol..

[10] Liu W., Pan Z., Zhao Y., Guo Y., Qiu S., Balasubramanian B., Jha R. (2022). Effects of heat stress on production performance, redox status, intestinal morphology and barrier-related gene expression, cecal microbiome, and metabolome in indigenous broiler chickens. Front. Physiol..

[11] Habashy W.S., Milfort M.C., Adomako K., Attia Y.A., Rekaya R., Aggrey S.E. (2017). Effect of heat stress on amino acid digestibility and transporters in meat-type chickens. Poult. Sci..

[12] Rostagno M.H. (2020). Effects of heat stress on the gut health of poultry. J. Anim. Sci..

[13] Zhang H., Majdeddin M., Gaublomme D., Taminiau B., Boone M., Elewaut D., Daube G., Josipovic I., Zhang K., Michiels J. (2021). 25-hydroxycholecalciferol reverses heat induced alterations in bone quality in finisher broilers associated with effects on intestinal integrity and inflammation. J. Anim. Sci. Biotechnol..

[14] Zhang H., Qin S., Zhu Y., Zhang X., Du P., Huang Y., Michiels J., Zeng Q., Chen W. (2022). Dietary resistant starch from potato regulates bone mass by modulating gut microbiota and concomitant short-chain fatty acids production in meat ducks. Front. Nutr..

[15] AOAC (2005). Official Methods of Analysis of AOAC International.

[16] Short F.J., Gorton P., Wiseman J., Boorman K.N. (1996). Determination of titanium dioxide added as an inert marker in chicken digestibility studies. Anim. Feed Sci. Technol..

[17] Zhang K.X., Zhang K.Y., Applegate T.J., Bai S.P., Ding X.M., Wang J.P., Peng H.W., Xuan Y., Su Z.W., Zeng Q.F. (2020). Evaluation of the standardized ileal digestibility of amino acids of rapeseed meals varying in protein solubility for Pekin ducks. Poult. Sci..

[18] Zeng X., Zhang K., Tian G., Ding X., Bai S., Wang J., Lv L., Liao Y., Xuan Y., Zeng Q. (2022). Effects of fat pre-emulsification on the growth performance, serum biochemical index, digestive enzyme activities, nutrient utilization, and standardized ileal digestibility of amino acids in Pekin ducks fed diets with different fat sources. Animals.

[19] SAS Institute Inc. (2016). SAS/STAT User’s Guide, Release 9.4.

[20] de Souza L.F.A., Espinha L.P., de Almeida E.A., Lunedo R., Furlan R.L., Macari M. (2016). How heat stress (continuous or cyclical) interferes with nutrient digestibility, energy and nitrogen balances and performance in broilers. Livest. Sci..

[21] Zhang S., Ou J., Luo Z., Kim I.H. (2020). Effect of dietary β-1, 3-glucan supplementation and heat stress on growth performance, nutrient digestibility, meat quality, organ weight, ileum microbiota, and immunity in broilers. Poult. Sci..

[22] Bonnet S., Geraert P.A., Lessire M., Carre B., Guillaumin S. (1997). Effect of high ambient temperature on feed digestibility in broilers. Poult. Sci..

[23] Wallis I.R., Balnave D. (1984). The influence of environmental temperature, age and sex on the digestibility of amino acids in growing broiler chickens. Br. Poult. Sci..

[24] Borin K., Lindberg J.E., Ogle R.B. (2006). Digestibility and digestive organ development in indigenous and improved chickens and ducks fed diets with increasing inclusion levels of cassava leaf meal. J. Anim. Physiol. Anim. Nutr..

[25] Kong C., Adeola O. (2013). Comparative amino acid digestibility for broiler chickens and White Pekin ducks. Poult. Sci..

[26] Cervantes M.M., Cota N., Arce G., Castillo E., Avelar S., Espinoza A. (2016). Morales. Effect of heat stress on performance and expression of selected amino acid and glucose transporters, HSP90, leptin and ghrelin in growing pigs. J. Therm. Biol..

[27] He J., He Y., Pan D., Cao J.X., Sun Y.Y., Zeng X. (2019). Associations of gut microbiota with heat stress-induced changes of growth, fat deposition, intestinal morphology, and antioxidant capacity in ducks. Front. Microbiol..

[28] Zhang C., Zhao X.H., Yang L., Chen X.Y., Jiang R.S., Jin S.H., Geng Z.Y. (2017). Resveratrol alleviates heat stress-induced impairment of intestinal morphology, microflora, and barrier integrity in broilers. Poult. Sci..

[29] Liu J., Teng P., Kim W.K., Applegate T.J. (2021). Assay considerations for fluorescein isothiocyanate-dextran (FITC-d): An indicator of intestinal permeability in broiler chickens. Poult. Sci..

[30] Gilani S., Howarth G.S., Nattrass G., Kitessa S.M., Barekatain R., Forder R., Tran C.D., Hughes R.J. (2018). Gene expression and morphological changes in the intestinal mucosa associated with increased permeability induced by short-term fasting in chickens. J. Anim. Physiol. Anim. Nutr..

[31] Koch F.U., Thom E., Albrecht R., Weikard W., Nolte B., Kuhla B., Kuehn C. (2019). Heat stress directly impairs gut integrity and recruits distinct immune cell populations into the bovine intestine. Proc. Natl. Acad. Sci. USA.

[32] Zhang H., Pertiwi H., Hou Y., Majdeddin M., Michiels J. (2024). Protective effects of Lactobacillus on heat stress-induced intestinal injury in finisher broilers by regulating gut microbiota and stimulating epithelial development. Sci. Total Environ..

[33] Patra A.K., Kar I. (2021). Heat stress on microbiota composition, barrier integrity, and nutrient transport in gut, production performance, and its amelioration in farm animals. J. Anim. Sci. Technol..

[34] Wang X.J., Feng J.H., Zhang M.H., Li X.M., Ma D.D., Chang S.S. (2018). Effects of high ambient temperature on the community structure and composition of ileal microbiome of broilers. Poult. Sci..

[35] Emami N.K., Schreier L.L., Greene E., Tabler T., Orlowski S.K., Anthony N.B., Proszkowiec-Weglarz M., Dridi S. (2022). Ileal microbial composition in genetically distinct chicken lines reared under normal or high ambient temperatures. Anim. Microbiome.

[36] Xing S., Wang X., Diao H., Zhang M., Zhou Y., Feng J. (2019). Changes in the cecal microbiota of laying hens during heat stress is mainly associated with reduced feed intake. Poult. Sci..

[37] Han S., Shin Y., Lee D., Kim K.M., Yang S., Kim D.S., Choi J., Lee S., Kim D. (2021). Lactobacillus rhamnosus HDB1258 modulates gut microbiota-mediated immune response in mice with or without lipopolysaccharide-induced systemic inflammation. BMC Microbiol..

[38] Munyaka P.M., Sepehri S., Ghia J., Khafipour E. (2016). Carrageenan gum and adherent invasive Escherichia coli in a piglet model of inflammatory bowel disease: Impact on intestinal mucosa-associated microbiota. Front. Microbiol..

[39] Huang Y., Wang Z., Ma H., Ji S., Chen Z., Cui Z., Chen J., Tang S. (2021). Dysbiosis and implication of the gut microbiota in diabetic retinopathy. Front. Cell. Infect. Microbiol..

[40] Al-Fataftah A., Abdelqader A. (2014). Effects of dietary Bacillus subtilis on heat-stressed broilers performance, intestinal morphology and microflora composition. Anim. Feed Sci. Technol..

